# Intersection of Religious Beliefs and Healthcare in Germany: A Guide to Integrating Religious Diversity in Medical Practice

**DOI:** 10.7759/cureus.82932

**Published:** 2025-04-24

**Authors:** Aysun Tekbaş, Arian Mauntel, Utz Settmacher, Teresa Festl-Wietek, Anne Herrmann-Werner, Bipin Doshi, Marie von Lilienfeld-Toal

**Affiliations:** 1 Department of General, Visceral and Vascular Surgery, Jena University Hospital, Friedrich Schiller University Jena, Jena, DEU; 2 “Advanced Clinician Scientist Programme”, Interdisciplinary Center of Clinical Research, Jena University Hospital, Friedrich Schiller University Jena, Jena, DEU; 3 Tuebingen Institute for Medical Education, Faculty of Medicine Tuebingen, Eberhard Karls University Tuebingen, Tuebingen, DEU; 4 Department of Internal Medicine VI/Psychosomatic Medicine and Psychotherapy, Tuebingen University Hospital, Tuebingen, DEU; 5 Department of Philosophy, University of Mumbai, Mumbai, IND; 6 Institute for Diversity Medicine, Ruhr-University-Bochum, Bochum, DEU

**Keywords:** culturally sensitive care, intercultural competencies, medical education, medical ethics, personalized medicine

## Abstract

Background

The increasing diversity in Germany’s population necessitates the application of culturally sensitive medical care. Religious and cultural beliefs significantly influence patient behavior and healthcare decisions. However, healthcare providers often lack knowledge in cultural beliefs related to medical treatment, particularly regarding minority religions such as Islam, the second-largest religious group in Germany. Addressing these gaps through medical education is essential for fostering intercultural competence and improving patient-centered care.

Objective

This pilot study assesses the knowledge of German medical students and a multi-faith expert group regarding Islamic-ethical principles in healthcare. Furthermore, expert interviews across multiple religions (Islam, Christianity, Judaism, Buddhism, Bahá'í, Jainism) explore additional faith-based considerations in clinical practice. In this context, the study aims to (1) identify knowledge gaps concerning religiously informed healthcare preferences, (2) generate foundational insights to inform medical education and promote culturally sensitive, patient-centered care, and (3) offer an insight into religious beliefs related to healthcare as an integral part of culture.

Methods

For this pilot study, a custom multiple-choice (MC) questionnaire on Islamic-ethical principles was administered to 50 medical students (ST) and an expert group (E, n = 6) comprising clinicians and religious authorities from the aforementioned faiths. Additionally, qualitative interviews were conducted with each expert to gain deeper insights into religion-specific considerations relevant to medical care.

Results

The MC questions on dietary regulations and gender relations were mostly answered correctly (84% and 92%, respectively). However, overall, both students and experts demonstrated limited knowledge of Islamic-ethical principles in healthcare. This was reflected in the fact that students did not answer any of the related questions with complete accuracy, while experts achieved fully accurate responses in only 50% of the cases. The results of the interviews were summarized, providing a detailed comparison of eight categories influencing medical treatment across Christianity, Islam, Buddhism, Judaism, Bahá'í, and Jainism.

Conclusion

Our study serves as an initial guide to fostering awareness and sensitivity in intercultural patient care in Germany. The complexity and diversity of interpretations within each faith, depending on personal and cultural preferences, have to be considered in personalized medicine.

## Introduction

The provision of healthcare to patients from diverse cultural backgrounds presents unique challenges to healthcare providers. When patients' cultural practices differ from those of the predominant majority in the respective country, healthcare professionals must navigate complex intersections of belief systems, values, and traditions to ensure effective and culturally sensitive care [1]. Effective communication is essential because misunderstandings can arise from unfamiliar practices, leading to incomplete medical history taking and, ultimately, incorrect treatment decisions [1, 2]. Cultural beliefs exert a profound influence on patients' health behaviors, treatment preferences, and healthcare decision-making processes. However, healthcare personnel frequently lack the necessary cultural competence and sensitivity towards the ethical complexities inherent in patient care [3]. This deficiency can manifest in various ways, including communication challenges and misunderstandings concerning hygiene rules, gender roles, acceptance of medication therapy, as well as perspectives on illness, treatment, death, and palliative care [4]. Patients, in turn, often experience uncertainty and anxiety due to unfamiliar processes in the medical environment. Additionally, they frequently feel disregarded [5].

From the perspective of care ethics, the relationship of responsibility and trust is seen as a fundamental aspect of the ethical obligation of healthcare professionals within the therapeutic team. Although the patient is generally best positioned to understand their own needs and preferences, they still require competent and compassionate support ("care") to articulate these needs and to take the necessary actions [6]. This principle becomes particularly important in complex and emotionally charged treatment settings, such as oncology, where cultural and religious convictions play a significant role in shaping patients' treatment decisions and outcomes [7].

Studies have highlighted the therapeutic value of religion and spirituality in empowering individuals facing oncological diseases, underscoring the importance of integrating these dimensions into holistic patient care [8-10]. López-Sierra et al. emphasize the role of religion and spirituality in mobilizing inner strengths and facilitating coping mechanisms for individuals with oncological diseases [10]. A profound understanding and respectful consideration of the patients’ beliefs can enhance the therapeutic relationship and sustainably improve treatment outcomes. This is especially relevant given that recent studies highlight instances of discrimination in the German healthcare system, impacting individuals from various backgrounds, including Muslims [5].

A case in point is the context of oncological surgery, where the integration of religious considerations is significant for Muslim patients. Here, preoperative preparation that involves collaboration between medical professionals, clergy, and family members is viewed as essential [8, 9]. Such an approach helps address patients’ specific spiritual and cultural needs, thereby improving both their surgical experience and postoperative outcomes.

Germany faces challenges in adequately addressing these aspects within its medical education sector [11]. However, the ongoing development of the National Competence-Based Learning Objectives Catalogue for Medicine (NKLM) highlights the growing scientific and political importance of addressing ethical, social, cultural, legal, and historical aspects in medical care [12]. Despite these developments, there remains a need for research that explores how future healthcare professionals engage with religious-ethical considerations in practice.

Against this backdrop, this study seeks to identify existing knowledge gaps regarding healthcare preferences shaped by religious beliefs, to generate initial insights that can inform medical training, and to underscore the importance of understanding religious beliefs as an essential component of culturally informed patient care. To achieve this, a dual-method strategy was employed: a focused assessment of Islamic-ethical principles through a custom-designed questionnaire, and a broader interreligious contextualization via expert interviews representing six faith traditions (Christianity, Islam, Judaism, Buddhism, Jainism, Bahá'í). Islam was selected as the focus of the questionnaire due to its significance as the second-largest religious group in Germany [13]. This approach allowed both depth in evaluating a relevant minority faith and breadth through expert contributions across traditions.

## Materials and methods

All analyses conducted are exploratory in nature, as this is a pilot study. Therefore, no formal sample size calculation was performed.

Expert interviews

Clinicians practicing the respective religions and clergy members (Christianity, Islam, Buddhism, Judaism, Jainism, and Bahá'í) were identified as potential experts for this study through a professional network of medical professionals and religious organizations. They were selected based on purposeful sampling, with the aim of ensuring representation of major religious traditions and feasibility of participation. They were then contacted via email or phone and provided with detailed information about the study. Participants responded to four open-ended questions, which were as follows:

(1) What regular religious obligations govern daily life? (2) What restrictions due to religion exist in social life? (3) What dietary rules could influence medical therapy? (4) What ethical principles play a role in illness and end-of-life decisions?

Participants submitted their responses either online in text format or verbally via telephone, allowing for detailed answers. Verbal responses were transcribed in real time. Following Mayring's methodology, the collected data were analyzed using a structured qualitative content analysis approach [14]. Based on the data, the text segments were categorized into emerging themes and coded to identify key categories.

Assessment of Islamic-ethical competences

A customized, internally developed multiple-choice questionnaire with six questions was implemented (Appendix A). The items were designed based on a focused literature review pertaining to intercultural competencies in medical practice [15-18], as well as existing teaching materials and seminar content related to religion and healthcare. Question 1 refers to basic knowledge about Islam, question 2 to end-of-life decisions, question 3 to religious practices during illness, question 4 to dietary regulations, question 5 to gender relations, and question 6 to organ transplantation.

The item construction followed established guidelines for multiple-choice question design, including clarity of wording, thematic relevance, and the use of plausible distractors. To ensure content validity, all items underwent a structured internal review process focusing on question quality and appropriate difficulty levels, particularly in view of the questionnaire’s use for assessing baseline knowledge. Due to the exploratory nature of the study, no formal psychometric validation (reliability testing, construct validation) was conducted. Each correct response was assigned one point, with a maximum total score of six.

Students from the Medical Faculty of Jena University, starting from the sixth semester, volunteered to participate (n = 50) and were given explicit instructions on how to complete the questionnaire. Their demographic information was collected, including age, gender, semester, and religious affiliation. For analytical purposes, religious affiliation was dichotomized into 'Islam' and 'Other,' with the latter encompassing all non-Islamic religious backgrounds. Furthermore, each expert completed the questionnaire. To safeguard confidentiality, all responses were anonymized.

Data analysis

Statistical analysis was performed with SPSS software (version 23, IBM Corp., Armonk, NY). Descriptive statistics were employed to summarize demographic information and frequencies. Due to the small sample size, Fisher’s exact test was applied to the questionnaire data to assess the significance of differences in the number of correct responses between students and experts.

## Results

Expert interviews

All four questions were thoroughly answered by the six experts. Following Mayring's method, the coded segments were categorized into eight distinct categories, each representing a religious practice relevant to healthcare. Furthermore, a comprehensive overview was compiled from the data:

Table 1 provides a detailed comparison of the eight categories influencing medical treatment across Christianity, Islam, Buddhism, Judaism, Bahá'í, and Jainism. However, it is important to note that these are individual opinions of the interviewed experts within our study. For all religions, denominations, and streams, there are often varied interpretations. The cultural context of each religion also frequently determines how religious practices are observed. It is therefore important to discuss with patients the significance of specific religious or ethical rules for their personal lives.

**Table 1:** Personal views from experts regarding aspects that may influence a patient's medical treatment in dependence of the respective religious affiliation. For non-prophetic religions, there are extremely diverse practices and philosophies. The information on Buddhism refers to the Soka Gakkai tradition (source: Soka Gakkai International Deutschland e.V.). Although Jainism represents a small minority in Germany, it is one of the major religious movements in India and is historically and culturally connected to Hinduism.

Category	Regular religious duties	Hygiene rules	Gender relations (cross-gender treatment)	Nutrition	Medication & bio-materials	View on illness, therapy & death	View on euthanasia & palliative therapy	Burial customs
Roman Catholic	• Sunday’s prayer obligation • Catholic clergy: daily Lauds and Vespers	• No specific religious hygiene rules	• Cross-gender treatment generally possible • Possibly cultural and denominational differences with a preference for same-gender interactions in clinical examinations	• No meat on Fridays and during the Lenten season, Monday to Saturday	• Typically, no regulations	• Illness and health as "ups and downs" • Prayers support healing • Death should be prepared for (e.g., anointing of the Sick) • Belief in life after death (paradise vs. hell)	• Assisted dying: rather restrictive • Palliative care: liberal attitude towards palliation • Emphasis on pain relief and care for the dying	• Since the Second Vatican Council, cremation and earth burial are possible
Sunni Islam	• Prayer 5 times/day • Fasting during the month of Ramadan (exceptions in special situations, such as illness, with implementation of compensation solutions)	• Adherence to rules for hygiene essential (circumcision, intimate and armpit shaving, washing of intimate areas after using toilet (with left hand), ritual washing and purity of textiles before prayer, full body washing after e.g. menstruation) • Food intake with right hand • Ritual washing of the deceased	• Restraint in physical and eye contact • Preference for same-gender medical treatment, possibly with the presence of a 3^rd^ person of the patient's gender (relatives, healthcare personnel); however, necessity relativizes commandments	• Allowed ("halal"): Consumption of islamically slaughtered cloven-hoofed animals (except pork), poultry, fish with scales and fins • Not allowed ("haram"): alcohol (and alcoholic products), consumption of non-islamically slaughtered animals, predators, and pigs	• In the absence of alternatives to medications containing alcohol and animal substances (from prohibited animals) as well as to biomaterials from, for example, pigs, these are allowed because: Necessity relativizes commandments	• Obligation to preserve health and care for the body (internally and externally) • Illness as a test from Allah • Death should be prepared(by oneself, relatives, clergy) for and accompanied (prayers, repentance for sins) • Belief in life after death (paradise vs. hell)	• Permitted: use of sedatives and painkillers without hastening death • "Letting die" (omission or reduction of treatment measures) if deemed appropriate by professional consensus • Not allowed: Direct active euthanasia, suicide, physician assistance to suicide	• Earth burial at the earliest possible time • Cremation/water burial not permitted • Preparation of the body for coffinless earth burial in a Muslim cemetery by washing, wrapping in a white cloth, funeral prayer • head of the deceased facing Mecca in the grave
Buddhism (Soka Gakkai)	• Daily practice (morning and evening Gongyo), study of Buddhism, participation in Buddhist activities (as self-motivated religious practice and for activating self-healing powers, not as an obligation)	• No specific rules	• No restrictions	• No restrictions (self-responsibility)	• No restrictions; all possibilities of modern medicine should be used for treating illnesses	• Birth, aging, illness, and death are the 4 fundamental sufferings of life; transforming the sufferings into 4 virtues of happiness, strong self, eternity, and purity by activating the Buddha nature (daily practice) • Health and illness are a unity and alternate in their manifestations • Illness is not a punishment; it can be the beginning of a spiritual quest • Life is eternal, life and death are a unity, death is both farewell and new beginning • karma "survives" death and remains in the next existence; crucial to change karma in this life • Praying for the deceased is an integral part of the daily practice	• Palliative medicine: everything that medicine offers to alleviate suffering and pain should be applied • Assisted dying: life is considered more precious than all the treasures of the universe and should be protected/preserved; individual decisions can be made if assisted dying is desired (self-responsibility and freedom of the individual) • Suicide should not be done; life is a treasure and karma cannot be changed by suicide (no condemnation if someone has committed suicide; rather prayers for those as they have been deeply desperate)	• Individual, no regulations
Liberal Judaism	• Obligation of Sabbath rest • Daily prayers	• In Orthodox Judaism, ritual immersion (Mikveh), for instance, after childbirth or menstruation	• Cross-gender treatment is generally possible • Preference for same-gender treatment or presence of a 3^rd^ person of the patient's gender (family member, healthcare personnel) among Orthodox Jews	• Adherence to Kashrut (religious dietary laws involving the separation of meat and dairy foods) • Allowed ("kosher"): bloodless meat from slaughtered cloven-hoofed animals that are also ruminants, poultry, and fish with scales and fins	• No restrictions in cases of medical necessity	• Health care and life-saving take precedence over religious duties • Obligation to seek medical treatment in case of illness	• Assisted dying and suicide assistance are prohibited • Suffering does not serve purification; pain and suffering must be alleviated • The dying process should not be artificially prolonged; it is accompanied as a natural process • Palliative sedation is allowed • Life is a gift from God; in doubt, always a decision for preserving life	• Ritual washing of the deceased (Tahara) and dressing immediately before burial • Earth burial at the earliest possible time, no cremation or water burial • Simple wooden coffin • Burial in a Jewish cemetery oriented toward Jerusalem, graves with perpetual rights, no time limit on the repose of the deceased, usually no exhumations
Bahá'í	• Daily prayer 1-3 times • Reading of holy scriptures and thanksgiving in the morning and evening • 19 days of fasting in March (often exempt for the sick) • Healing prayers	• Face and hand washing before daily prayer	• Cross-gender treatment allowed	• Free to choose, but predominantly plant-based diet recommended • Alcohol only for medical reasons allowed • Diets recommended	• Diets, healthy foods, and the application of water showers are recommended preventively and for treatment • Regarding medications, advice of competent physicians should be followed	• Doctor's visit with adherence to recommendations strongly recommended in case of illness • Illness can be a trial, but initially a task for the patient and the physician • Healing prayers recommended • Health considered one of the recommended life tasks (including physical exercise) • Facing death with "hope and expectation"	• Palliative therapy is strongly advocated as reducing suffering is a task of medicine • If a person truly wishes to die, he/she should implore God for it • For suicides, believers should "pray a lot"	• Washing, wrapping in 1-5 silk or cotton cloths, engraved death ring on the finger • Earth burial with face in the direction of prayer (Akka), as soon as possible in a solid coffin, maximum 1 hour's journey from the place of death • Funeral prayer at burial
Jainism	• Many religious rituals, but flexible in implementation • Boiled water can be consumed during fasting; thus, the intake of medication is also possible (however, with subsequent "repentance")	• Body and mouth cleansing before religious rituals/worship (body with a damp cloth; principle: no wastage of water) • Selection of the cleanest possible clothing (must not be washed) during worship • A mask should be worn when touching the figures	• Difference between monks/nuns and ordinary believers • Monks and nuns: no cross-gender treatment; if necessary, the respective "guru" must prescribe a "repentance ritual" • Ordinary believers: cross-gender treatment allowed	• Strictly vegetarian • Prohibition of underground-growing foods (onion, potato, etc.), alcohol, honey, eggs • No eating after sunset (intermittent fasting)	• Allowed in the absence of alternatives to medications containing alcohol and animal substances in vital necessity (with subsequent "repentance") • Monks and nuns are not allowed to ingest alcoholic or animal substances even in vital threat (suffering and dying are accepted); however, this is sometimes not strictly practiced, and the "repentance ritual" is also employed here	• Illness is the cause of "karma" (karma of "previous and present lives") • Everything that happens in life is an expression of karma and one's own efforts (thus in the hands of the individual) • Maintenance of health and "non-violent" treatment are important • Lifespan determined in "previous life," can be slightly modified • Death is the beginning of a new life (rebirth)	• Patient can decide to cease all therapies and food intake in case of severe incurable illness ("sallekhana": spiritual, voluntary death) • During "sallekhana," patients are honored and worshipped, death is celebrated • Suicide or euthanasia not allowed	• Cremation

The expert with knowledge of the Roman Catholic faith stated that religious duties such as Sunday prayer are obligatory, while also emphasizing practices like fasting and the anointing of the sick.

Islam (Sunni) emphasizes hygiene rules, with practices such as ritual washing before prayer and specific dietary restrictions like consuming halal food. Medical treatment in Islam is guided by principles of necessity and preserving health, with a preference for same-gender medical care when possible.

The interview partner practicing Buddhism (Soka Gakkai) emphasized the unity of health and illness, viewing suffering as part of life's fundamental experiences. Buddhist perspectives on euthanasia stress individual autonomy and the sanctity of life.

Judaism (Liberal) highlights religious practices such as Sabbath rest and dietary laws, with a strong emphasis on preserving life and seeking medical treatment when necessary. Burial customs involve rituals like Tahara (ritual washing) and earth burial in Jewish cemeteries.

Bahá'í teachings include daily prayers, fasting, and adherence to a predominantly plant-based diet. Medical treatment is guided by principles of seeking competent medical advice and facing death with hope and expectation.

The Jain scholar highlighted principles of non-violence and karma, with strict dietary restrictions and rituals like fasting. Jain perspectives on medical treatment involve balancing religious principles with medical necessity, with options like voluntary spiritual death (sallekhana) available for incurable illnesses.

Overall, the overview highlights the importance of understanding and respecting patients' religious beliefs and practices in healthcare settings.

Islamic-ethical competences of medical students based on a custom-designed questionnaire (Appendix A)

Table 2 presents the demographic characteristics of the 50 students who took part in the survey. A substantial proportion, comprising 31 individuals (62 %), were enrolled in their 6th semester (third year). The age of the majority of students ranged between 20 and 25 years (n = 46, 92 %). Of the respondents, 72% (n=36) were female. Regarding religious affiliation, only one (2%) identified as Muslim. Within this student population, a small fraction, 4% (n=2), managed to answer only one or two questions accurately. Around 24% (n=12) of students answered three questions, and the majority of students, 38% (n = 19), answered four questions correctly. Around 16% (n=8) got five questions right, and only 14% (n=7) answered all questions correctly. Table 3 shows the number of accurate responses each question received. All questions, except for question 6 (36%), were answered correctly by more than 50% of the students. Notably, there appears to be a strong understanding of dietary regulations and gender relations (questions 4 (84%) and 5 (92%)).

**Table 2:** Demographic data of n = 50 students. m = male, f = female

Parameter	%	n
Gender		
Male	26	13
Female	72	36
Non-binary	2	1
Semester		
6^th^	62	31
7^th^	14	7
8^th^	4	2
9^th^	14	7
≥10^th^	6	3
Age (years)		
20 - 25	92	46
26 – 30	6	3
≥30	2	1
Religious affiliation		
Islam	2	1
Other	98	49

Islamic-ethical competences of experts and comparison with students

Based on the absolute values, the experts performed better than the students on all questions except for question 3 (religious practices). While no questions were answered correctly by 100% of the students, the experts achieved 100% correct answers on questions 1 (basic knowledge), 4 (dietary regulations), and 5 (gender relations). However, the difference between the groups was not statistically significant (p > 0.05) (Table 3).

**Table 3:** Number of correct answers each question received by students and experts with the respective p-value. The questions are listed in the appendix.

Question no	Correctly answered by students	Correctly answered by experts	p-value
1 (basic knowledge)	29/50 (58 %)	6/6 (100 %)	0.074
2 (end-of-life decisions)	30/50 (60 %)	5/6 (83 %)	0.393
3 (practices in illness)	35/50 (70 %)	3/6 (50 %)	0.374
4 (dietary regulations)	42/50 (84 %)	6/6 (100 %)	0.578
5 (gender relations)	46/50 (92 %)	6/6 (100 %)	1.0
6 (organ transplantation)	18/50 (36 %)	4/6 (67%)	0.198

## Discussion

Our study provides insights into how various religious affiliations - Christianity, Islam, Buddhism, Judaism, Bahá'í, and Jainism - affect medical treatment, as well as the level of understanding among experts and medical students regarding Islamic ethical aspects of medicine. With this, the aim is to contribute to more personalized and empathetic medical care. However, it is important to note that the complexity and diversity of interpretations within each faith can vary based on personal and cultural preferences. Our overview serves in this context as a guide to support personal conversations with patients.

According to the results of the survey, key areas of focus regarding Islamic-ethical principles are dietary regulations and gender relations. This may be due to the fact that such topics are often covered in the media and, therefore, accessible to the wider population. However, the finding that only 14% of the students answered all the questions pertinent to patient clinical care correctly underscores a significant need for enhanced education, particularly in less publicly discussed but clinically relevant areas of Islamic ethics. This includes topics such as end-of-life decisions, organ donation, and confidentiality, which were more often answered incorrectly. Therefore, the knowledge gap should be understood as topic-specific rather than a general lack of awareness.

Our endeavor seeks to promote a healthcare environment characterized by inclusivity, empathy, and culturally competent care, thereby fostering holistic well-being and healing for all patients, irrespective of their religious backgrounds. By recognizing and respecting patients' diverse religious beliefs and practices, healthcare professionals can foster trust, enhance communication, and ultimately deliver more tailored and effective care [19, 20]. Culturally sensitive approaches not only acknowledge patients' individual identities but also contribute to improved patient satisfaction, treatment adherence, and health outcomes [19, 21]. From the perspective of care ethics, an empathetic approach allows the patient to directly express their specific wishes regarding aspects of their medical treatment [6].

However, incorporating cultural competence education into medical school curricula faces substantial obstacles, such as limited resources, time constraints, and competing priorities within the educational framework. Overcoming these challenges necessitates a greater exchange of experiences and best practices among European medical schools [22].

In our study, potential biases were present in the selection of participants for expert interviews, stemming from various factors such as convenience sampling, accessibility to experts within our professional networks, and potential cultural biases inherent in the selection process. While efforts were made to include a diverse range of religious perspectives, it's crucial to acknowledge the potential limitations in the representativeness of our expert sample. Given the small sample, the responses should be seen as a preliminary orientation rather than a definitive benchmark. Nonetheless, the most prevalent religious affiliations in the medical context have been effectively represented, thereby amplifying patients' voices. This is especially pertinent given the findings of the NaDiRa Report 2023 (National Discrimination and Racism Monitor) “Racism and its symptoms”, which surveyed 21,000 individuals in Germany about their experiences with racism [5]. The report found that 35% of Muslim women experienced unfair treatment by medical staff, with the figures increasing to 39% for Black women and 29% for Asian women. Furthermore, 13-14% of Black, Asian, and Muslim women, along with 8% of men from these groups, have avoided or postponed medical treatment due to concerns about discrimination [5]. In light of this information, the overview we compiled from expert interviews presents religious-ethical aspects related to healthcare contribute to the practical improvement of interactions with people from diverse cultural backgrounds in the healthcare system.

A further limitation of the study is the lack of a detailed validation process for the internally developed questionnaire. Although the questions were based on literature review and teaching content, we did not conduct formal pilot testing or statistical validation. No reliability metrics were calculated, limiting our ability to assess the consistency and robustness of the questionnaire. This methodological shortcoming affects the strength of the conclusions that can be drawn and highlights the need for future studies to develop and validate more rigorously tested assessment tools. 

Despite these limitations, we see our study as an initial step that highlights both the need and the potential for enhanced religious-ethical competence in medical education. A further step involves developing a teaching framework to integrate these topics into the curriculum.

## Conclusions

Our study highlights the importance of religious competence in medical practice, emphasizing the need for healthcare professionals to understand the ethical and spiritual dimensions of patient care. Islamic medical ethics, as an example of intercultural competence, includes aspects such as dietary regulations and gender relations, which are widely recognized. However, our findings reveal a significant gap in comprehensive knowledge among medical students. To address this, we provided an overview of religious-ethical perspectives on healthcare across six major religions. While this overview may offer a useful reference for educational contexts, implications for curricular development require further evaluation. Therefore, our study serves as an initial guide for integrating religious-ethical perspectives into educational efforts aimed at better preparing healthcare professionals to provide compassionate and culturally competent care to diverse patient populations.

## Appendices

Appendix A

Multiple-choice questions on Islamic ethical principles for assessing students' knowledge.

1. Which aspect belongs to the „5 pillars of Islam“ (1 answer combination):

A. The Declaration of Faith.

B. Praying 5 times a day.

C. Fasting during the month of Ramadan.

D. The pilgrimage to Mecca.

E. Almsgiving (2.5% of wealth annually).

- Only answers A and B are correct.

- Only answers A and D are correct.

- Only answers B and C are correct.

- All answers are correct except for answer E.

- All answers are correct.

2. Which statement about accompanying dying Muslim patients is correct? (1 option)

A. Active euthanasia is allowed.

B. The use of sedatives and painkillers is permitted without hastening death.

C. End-of-life care has no significance in Islam.

D. There are widespread Muslim chaplains in Germany.

E. End-of-life care must be conducted by a religious leader.

3. Which statements are correct? (1 answer combination)

A. Sick individuals are not required to fast during the month of Ramadan.

B. Patients in the hospital are not required to pray.

C. Efforts should be made to seek alternatives to gelatin- and alcohol-containing medications.

- Only answers A and B are correct.

- Only answer A is correct.

- Only answers B and C are correct.

- All answers are correct.

 - Only answers A and C are correct.

4. What is understood by the principle of "halal" regarding nutrition in Islam? (1 option)

A. No meat may be consumed.

B. Consumption of ritually slaughtered animals, except for pork, is permitted.

C. Seafood may not be consumed.

D. Consumption of alcohol in small quantities is permitted.

E. No answer is correct.

5. How should the physician or nursing staff behave in situations involving cross-gender examinations with Muslim patients? Which of the following statements is correct?

A. A same-gender examination is desirable.

B. Cross-gender examinations are not allowed even in emergencies.

C. Women cannot be examined by men.

D. The examination should be postponed to a day when treatment can be provided by a healthcare professional of the patient's gender.

- Only answer A is correct.

- All answers are correct.

- Only answers B and C are correct.

- Only answers B, C, and D are correct.

- All answers are incorrect.

6. What significance does organ donation hold in Islam? Which of the following statements is correct?

A. Organ donation is encouraged once a certain age is reached.

B. Organ donation plays a rather minor role in Islam.

C. Animal-derived materials, such as pig heart valves, cannot be transplanted.

D. With a medical indication, transplantation can be performed while adhering to certain regulations.

- Only answer A is correct.

- Only answer D is correct.

- Only answers A, B, and C are correct.

- Only answers B, C, and D are correct.

- All answers are incorrect.
